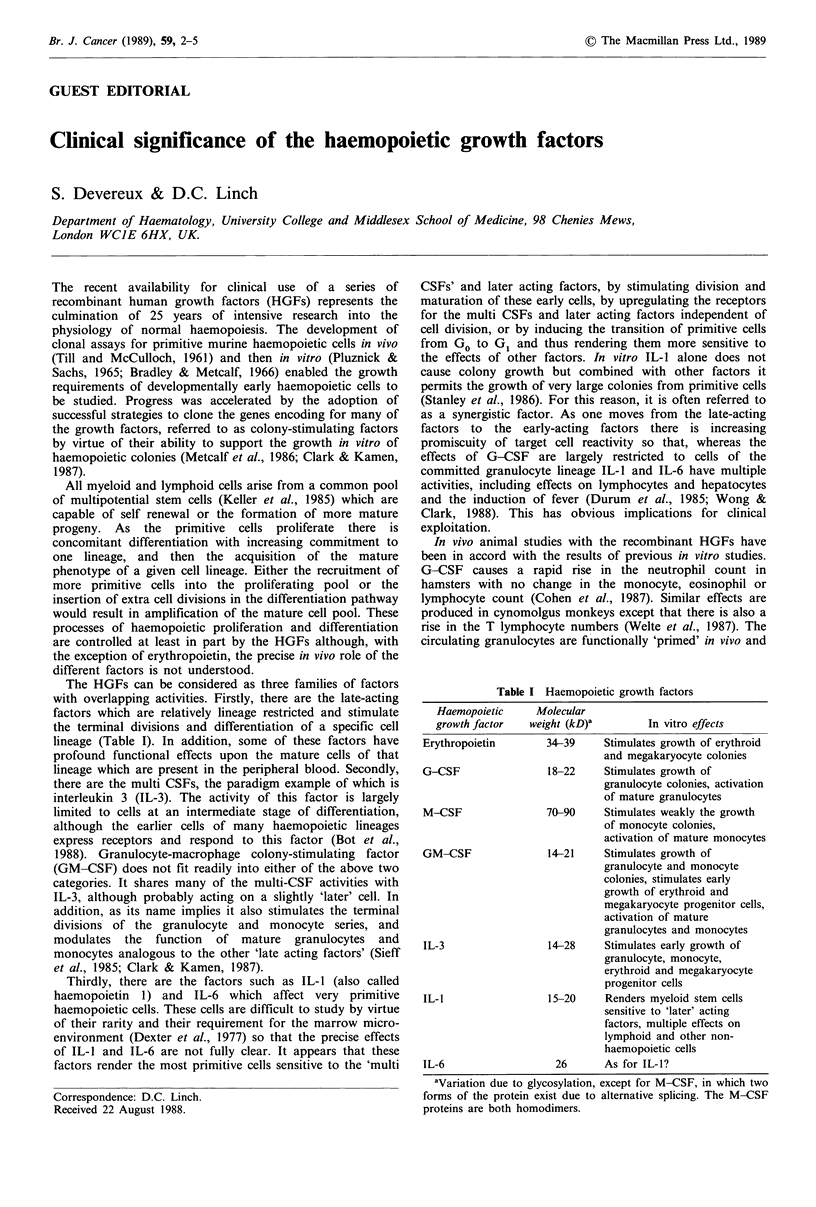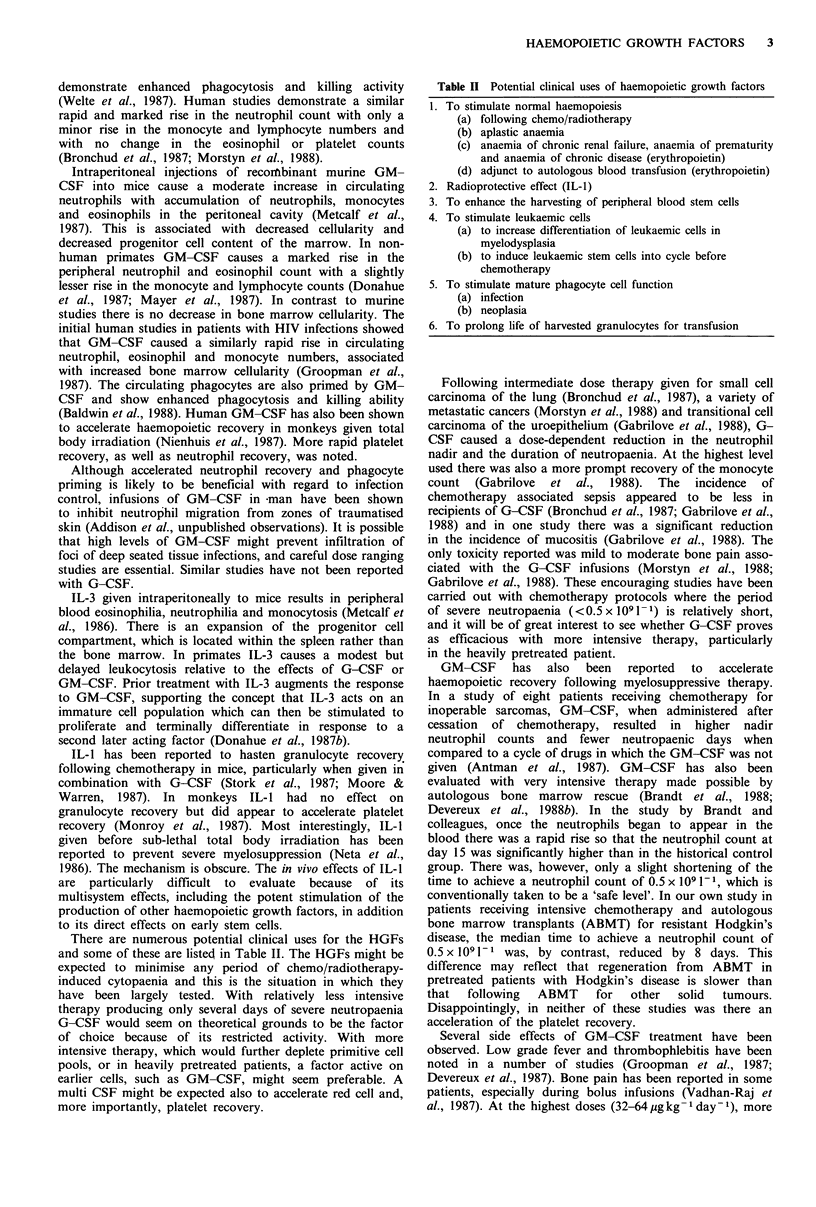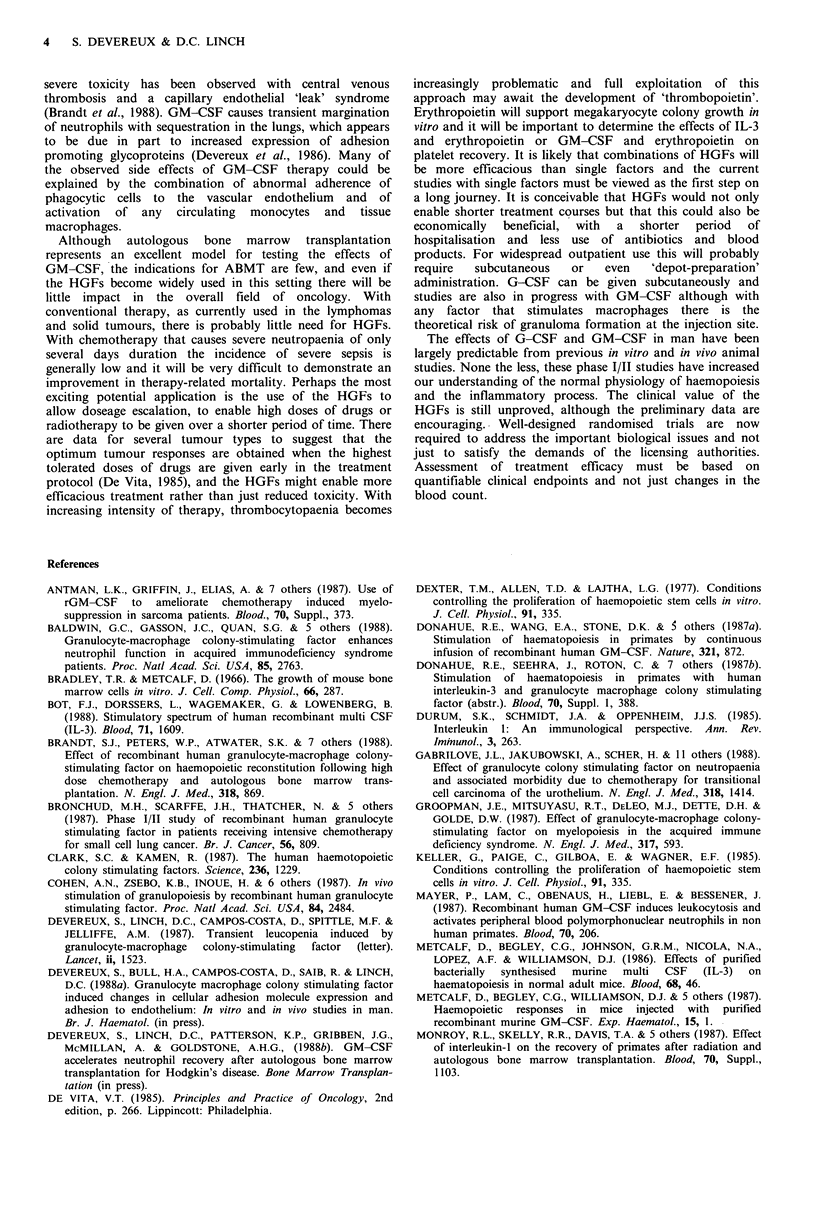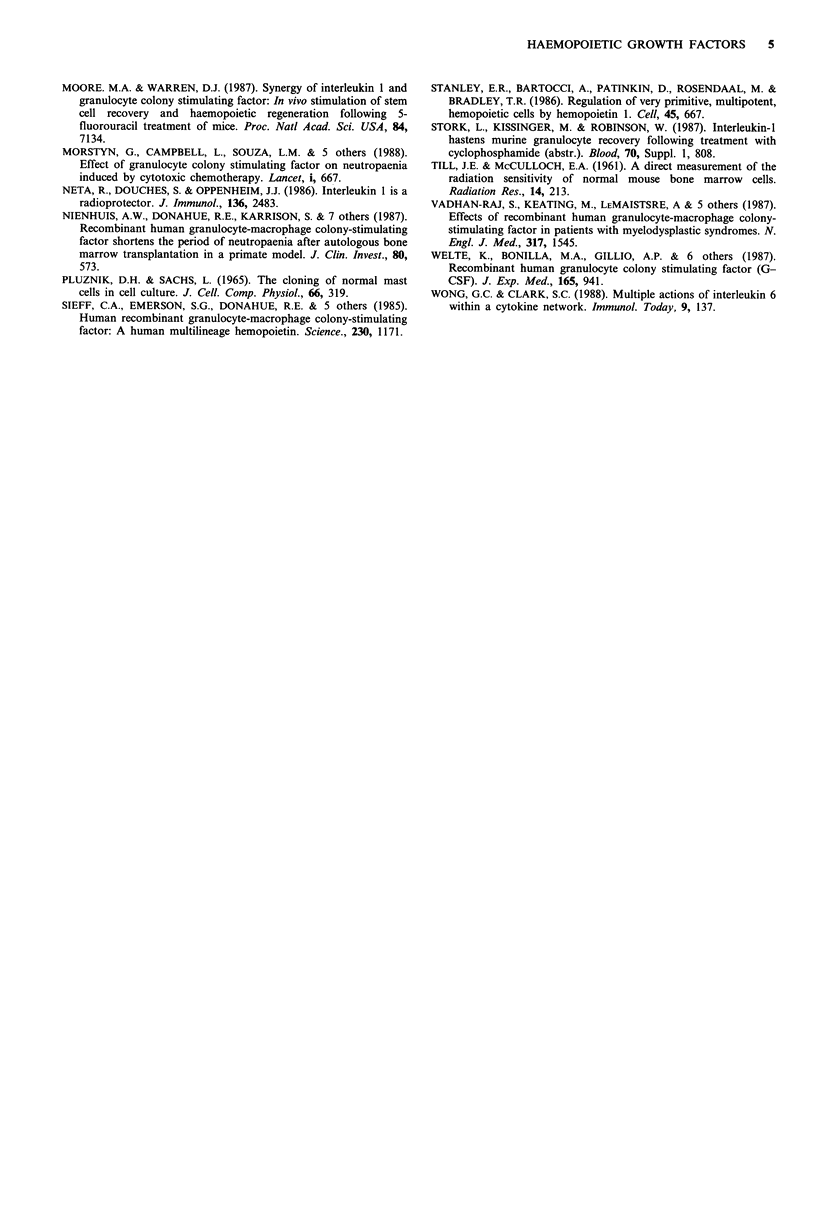# Clinical significance of the haemopoietic growth factors.

**DOI:** 10.1038/bjc.1989.2

**Published:** 1989-01

**Authors:** S. Devereux, D. C. Linch

**Affiliations:** Department of Haematology, University College and Middlesex School of Medicine, London, UK.


Br. J. Cancer (1989), 59, 2–5 © The Macmillan Press Ltd., 1989

GUEST EDITORIAL

### Clinical significance of the haemopoietic growth factors

S. Devereux & D.C. Linch

*Department of Haematology, University College and Middlesex School of Medicine, 98 Chenies Mews, London WC1E 6HX, UK.*

The recent availability for clinical use of a series of recombinant human growth factors (HGFs) represents the culmination of 25 years of intensive research into the physiology of normal haemopoiesis. The development of clonal assays for primitive murine haemopoietic cells *in vivo* (Till and McCulloch, 1961) and then *in vitro* (Pluznick & Sachs, 1965; Bradley & Metcalf, 1966) enabled the growth requirements of developmentally early haemopoietic cells to be studied. Progress was accelerated by the adoption of successful strategies to clone the genes encoding for many of the growth factors, referred to as colony-stimulating factors by virtue of their ability to support the growth *in vitro* of haemopoietic colonies (Metcalf *et al.*, 1986; Clark & Kamen, 1987).

All myeloid and lymphoid cells arise from a common pool of multipotential stem cells (Keller *et al.*, 1985) which are capable of self renewal or the formation of more mature progeny. As the primitive cells proliferate there is concomitant differentiation with increasing commitment to one lineage, and then the acquisition of the mature phenotype of a given cell lineage. Either the recruitment of more primitive cells into the proliferating pool or the insertion of extra cell divisions in the differentiation pathway would result in amplification of the mature cell pool. These processes of haemopoietic proliferation and differentiation are controlled at least in part by the HGFs although, with the exception of erythropoietin, the precise *in vivo* role of the different factors is not understood.

The HGFs can be considered as three families of factors with overlapping activities. Firstly, there are the late-acting factors which are relatively lineage restricted and stimulate the terminal divisions and differentiation of a specific cell lineage (Table I). In addition, some of these factors have profound functional effects upon the mature cells of that lineage which are present in the peripheral blood. Secondly, there are the multi CSFs, the paradigm example of which is interleukin 3 (IL-3). The activity of this factor is largely limited to cells at an intermediate stage of differentiation, although the earlier cells of many haemopoietic lineages express receptors and respond to this factor (Bot *et al.*, 1988). Granulocyte-macrophage colony-stimulating factor (GM–CSF) does not fit readily into either of the above two categories. It shares many of the multi-CSF activities with IL-3, although probably acting on a slightly 'later' cell. In addition, as its name implies it also stimulates the terminal divisions of the granulocyte and monocyte series, and modulates the function of mature granulocytes and monocytes analogous to the other 'late acting factors' (Sieff *et al.*, 1985; Clark & Kamen, 1987).

Thirdly, there are the factors such as IL-1 (also called haemopoietin 1) and IL-6 which affect very primitive haemopoietic cells. These cells are difficult to study by virtue of their rarity and their requirement for the marrow micro-environment (Dexter *et al.*, 1977) so that the precise effects of IL-1 and IL-6 are not fully clear. It appears that these factors render the most primitive cells sensitive to the 'multi CSFs' and later acting factors, by stimulating division and maturation of these early cells, by upregulating the receptors for the multi CSFs and later acting factors independent of cell division, or by inducing the transition of primitive cells from $G_0$ to $G_1$ and thus rendering them more sensitive to the effects of other factors. *In vitro* IL-1 alone does not cause colony growth but combined with other factors it permits the growth of very large colonies from primitive cells (Stanley *et al.*, 1986). For this reason, it is often referred to as a synergistic factor. As one moves from the late-acting factors to the early-acting factors there is increasing promiscuity of target cell reactivity so that, whereas the effects of G–CSF are largely restricted to cells of the committed granulocyte lineage IL-1 and IL-6 have multiple activities, including effects on lymphocytes and hepatocytes and the induction of fever (Durum *et al.*, 1985; Wong & Clark, 1988). This has obvious implications for clinical exploitation.

*In vivo* animal studies with the recombinant HGFs have been in accord with the results of previous *in vitro* studies. G–CSF causes a rapid rise in the neutrophil count in hamsters with no change in the monocyte, eosinophil or lymphocyte count (Cohen *et al.*, 1987). Similar effects are produced in cynomolgus monkeys except that there is also a rise in the T lymphocyte numbers (Welte *et al.*, 1987). The circulating granulocytes are functionally 'primed' *in vivo* and

**Table I** Haemopoietic growth factors

| *Haemopoietic growth factor* | *Molecular weight (kD)*[a] | In vitro *effects* |
|---|---|---|
| Erythropoietin | 34–39 | Stimulates growth of erythroid and megakaryocyte colonies |
| G–CSF | 18–22 | Stimulates growth of granulocyte colonies, activation of mature granulocytes |
| M–CSF | 70–90 | Stimulates weakly the growth of monocyte colonies, activation of mature monocytes |
| GM–CSF | 14–21 | Stimulates growth of granulocyte and monocyte colonies, stimulates early growth of erythroid and megakaryocyte progenitor cells, activation of mature granulocytes and monocytes |
| IL-3 | 14–28 | Stimulates early growth of granulocyte, monocyte, erythroid and megakaryocyte progenitor cells |
| IL-1 | 15–20 | Renders myeloid stem cells sensitive to 'later' acting factors, multiple effects on lymphoid and other non-haemopoietic cells |
| IL-6 | 26 | As for IL-1? |

[a]Variation due to glycosylation, except for M–CSF, in which two forms of the protein exist due to alternative splicing. The M–CSF proteins are both homodimers.

Correspondence: D.C. Linch.
Received 22 August 1988.

demonstrate enhanced phagocytosis and killing activity (Welte *et al.*, 1987). Human studies demonstrate a similar rapid and marked rise in the neutrophil count with only a minor rise in the monocyte and lymphocyte numbers and with no change in the eosinophil or platelet counts (Bronchud *et al.*, 1987; Morstyn *et al.*, 1988).

Intraperitoneal injections of recombinant murine GM–CSF into mice cause a moderate increase in circulating neutrophils with accumulation of neutrophils, monocytes and eosinophils in the peritoneal cavity (Metcalf *et al.*, 1987). This is associated with decreased cellularity and decreased progenitor cell content of the marrow. In non-human primates GM–CSF causes a marked rise in the peripheral neutrophil and eosinophil count with a slightly lesser rise in the monocyte and lymphocyte counts (Donahue *et al.*, 1987; Mayer *et al.*, 1987). In contrast to murine studies there is no decrease in bone marrow cellularity. The initial human studies in patients with HIV infections showed that GM–CSF caused a similarly rapid rise in circulating neutrophil, eosinophil and monocyte numbers, associated with increased bone marrow cellularity (Groopman *et al.*, 1987). The circulating phagocytes are also primed by GM–CSF and show enhanced phagocytosis and killing ability (Baldwin *et al.*, 1988). Human GM–CSF has also been shown to accelerate haemopoietic recovery in monkeys given total body irradiation (Nienhuis *et al.*, 1987). More rapid platelet recovery, as well as neutrophil recovery, was noted.

Although accelerated neutrophil recovery and phagocyte priming is likely to be beneficial with regard to infection control, infusions of GM–CSF in man have been shown to inhibit neutrophil migration from zones of traumatised skin (Addison *et al.*, unpublished observations). It is possible that high levels of GM–CSF might prevent infiltration of foci of deep seated tissue infections, and careful dose ranging studies are essential. Similar studies have not been reported with G–CSF.

IL-3 given intraperitoneally to mice results in peripheral blood eosinophilia, neutrophilia and monocytosis (Metcalf *et al.*, 1986). There is an expansion of the progenitor cell compartment, which is located within the spleen rather than the bone marrow. In primates IL-3 causes a modest but delayed leukocytosis relative to the effects of G–CSF or GM–CSF. Prior treatment with IL-3 augments the response to GM–CSF, supporting the concept that IL-3 acts on an immature cell population which can then be stimulated to proliferate and terminally differentiate in response to a second later acting factor (Donahue *et al.*, 1987*b*).

IL-1 has been reported to hasten granulocyte recovery following chemotherapy in mice, particularly when given in combination with G–CSF (Stork *et al.*, 1987; Moore & Warren, 1987). In monkeys IL-1 had no effect on granulocyte recovery but did appear to accelerate platelet recovery (Monroy *et al.*, 1987). Most interestingly, IL-1 given before sub-lethal total body irradiation has been reported to prevent severe myelosuppression (Neta *et al.*, 1986). The mechanism is obscure. The *in vivo* effects of IL-1 are particularly difficult to evaluate because of its multisystem effects, including the potent stimulation of the production of other haemopoietic growth factors, in addition to its direct effects on early stem cells.

There are numerous potential clinical uses for the HGFs and some of these are listed in Table II. The HGFs might be expected to minimise any period of chemo/radiotherapy-induced cytopaenia and this is the situation in which they have been largely tested. With relatively less intensive therapy producing only several days of severe neutropaenia G–CSF would seem on theoretical grounds to be the factor of choice because of its restricted activity. With more intensive therapy, which would further deplete primitive cell pools, or in heavily pretreated patients, a factor active on earlier cells, such as GM–CSF, might seem preferable. A multi CSF might be expected also to accelerate red cell and, more importantly, platelet recovery.

**Table II** Potential clinical uses of haemopoietic growth factors

1. To stimulate normal haemopoiesis
   (a) following chemo/radiotherapy
   (b) aplastic anaemia
   (c) anaemia of chronic renal failure, anaemia of prematurity and anaemia of chronic disease (erythropoietin)
   (d) adjunct to autologous blood transfusion (erythropoietin)
2. Radioprotective effect (IL-1)
3. To enhance the harvesting of peripheral blood stem cells
4. To stimulate leukaemic cells
   (a) to increase differentiation of leukaemic cells in myelodysplasia
   (b) to induce leukaemic stem cells into cycle before chemotherapy
5. To stimulate mature phagocyte cell function
   (a) infection
   (b) neoplasia
6. To prolong life of harvested granulocytes for transfusion

Following intermediate dose therapy given for small cell carcinoma of the lung (Bronchud *et al.*, 1987), a variety of metastatic cancers (Morstyn *et al.*, 1988) and transitional cell carcinoma of the uroepithelium (Gabrilove *et al.*, 1988), G–CSF caused a dose-dependent reduction in the neutrophil nadir and the duration of neutropaenia. At the highest level used there was also a more prompt recovery of the monocyte count (Gabrilove *et al.*, 1988). The incidence of chemotherapy associated sepsis appeared to be less in recipients of G–CSF (Bronchud *et al.*, 1987; Gabrilove *et al.*, 1988) and in one study there was a significant reduction in the incidence of mucositis (Gabrilove *et al.*, 1988). The only toxicity reported was mild to moderate bone pain associated with the G–CSF infusions (Morstyn *et al.*, 1988; Gabrilove *et al.*, 1988). These encouraging studies have been carried out with chemotherapy protocols where the period of severe neutropaenia ($<0.5 \times 10^9 l^{-1}$) is relatively short, and it will be of great interest to see whether G–CSF proves as efficacious with more intensive therapy, particularly in the heavily pretreated patient.

GM–CSF has also been reported to accelerate haemopoietic recovery following myelosuppressive therapy. In a study of eight patients receiving chemotherapy for inoperable sarcomas, GM–CSF, when administered after cessation of chemotherapy, resulted in higher nadir neutrophil counts and fewer neutropaenic days when compared to a cycle of drugs in which the GM–CSF was not given (Antman *et al.*, 1987). GM–CSF has also been evaluated with very intensive therapy made possible by autologous bone marrow rescue (Brandt *et al.*, 1988; Devereux *et al.*, 1988*b*). In the study by Brandt and colleagues, once the neutrophils began to appear in the blood there was a rapid rise so that the neutrophil count at day 15 was significantly higher than in the historical control group. There was, however, only a slight shortening of the time to achieve a neutrophil count of $0.5 \times 10^9 l^{-1}$, which is conventionally taken to be a 'safe level'. In our own study in patients receiving intensive chemotherapy and autologous bone marrow transplants (ABMT) for resistant Hodgkin's disease, the median time to achieve a neutrophil count of $0.5 \times 10^9 l^{-1}$ was, by contrast, reduced by 8 days. This difference may reflect that regeneration from ABMT in pretreated patients with Hodgkin's disease is slower than that following ABMT for other solid tumours. Disappointingly, in neither of these studies was there an acceleration of the platelet recovery.

Several side effects of GM–CSF treatment have been observed. Low grade fever and thrombophlebitis have been noted in a number of studies (Groopman *et al.*, 1987; Devereux *et al.*, 1987). Bone pain has been reported in some patients, especially during bolus infusions (Vadhan-Raj *et al.*, 1987). At the highest doses (32–64 $\mu g\, kg^{-1}\, day^{-1}$), more severe toxicity has been observed with central venous thrombosis and a capillary endothelial 'leak' syndrome (Brandt *et al.*, 1988). GM–CSF causes transient margination of neutrophils with sequestration in the lungs, which appears to be due in part to increased expression of adhesion promoting glycoproteins (Devereux *et al.*, 1986). Many of the observed side effects of GM–CSF therapy could be explained by the combination of abnormal adherence of phagocytic cells to the vascular endothelium and of activation of any circulating monocytes and tissue macrophages.

Although autologous bone marrow transplantation represents an excellent model for testing the effects of GM–CSF, the indications for ABMT are few, and even if the HGFs become widely used in this setting there will be little impact in the overall field of oncology. With conventional therapy, as currently used in the lymphomas and solid tumours, there is probably little need for HGFs. With chemotherapy that causes severe neutropaenia of only several days duration the incidence of severe sepsis is generally low and it will be very difficult to demonstrate an improvement in therapy-related mortality. Perhaps the most exciting potential application is the use of the HGFs to allow doseage escalation, to enable high doses of drugs or radiotherapy to be given over a shorter period of time. There are data for several tumour types to suggest that the optimum tumour responses are obtained when the highest tolerated doses of drugs are given early in the treatment protocol (De Vita, 1985), and the HGFs might enable more efficacious treatment rather than just reduced toxicity. With increasing intensity of therapy, thrombocytopaenia becomes increasingly problematic and full exploitation of this approach may await the development of 'thrombopoietin'. Erythropoietin will support megakaryocyte colony growth *in vitro* and it will be important to determine the effects of IL-3 and erythropoietin or GM–CSF and erythropoietin on platelet recovery. It is likely that combinations of HGFs will be more efficacious than single factors and the current studies with single factors must be viewed as the first step on a long journey. It is conceivable that HGFs would not only enable shorter treatment courses but that this could also be economically beneficial, with a shorter period of hospitalisation and less use of antibiotics and blood products. For widespread outpatient use this will probably require subcutaneous or even 'depot-preparation' administration. G–CSF can be given subcutaneously and studies are also in progress with GM–CSF although with any factor that stimulates macrophages there is the theoretical risk of granuloma formation at the injection site.

The effects of G–CSF and GM–CSF in man have been largely predictable from previous *in vitro* and *in vivo* animal studies. None the less, these phase I/II studies have increased our understanding of the normal physiology of haemopoiesis and the inflammatory process. The clinical value of the HGFs is still unproved, although the preliminary data are encouraging. Well-designed randomised trials are now required to address the important biological issues and not just to satisfy the demands of the licensing authorities. Assessment of treatment efficacy must be based on quantifiable clinical endpoints and not just changes in the blood count.

### References

ANTMAN, L.K., GRIFFIN, J., ELIAS, A. & 7 others (1987). Use of rGM–CSF to ameliorate chemotherapy induced myelo-suppression in sarcoma patients. *Blood.*, **70**, Suppl., 373.

BALDWIN, G.C., GASSON, J.C., QUAN, S.G. & 5 others (1988). Granulocyte-macrophage colony-stimulating factor enhances neutrophil function in acquired immunodeficiency syndrome patients. *Proc. Natl Acad. Sci. USA*, **85**, 2763.

BRADLEY, T.R. & METCALF, D. (1966). The growth of mouse bone marrow cells *in vitro*. *J. Cell. Comp. Physiol.*, **66**, 287.

BOT, F.J., DORSSERS, L., WAGEMAKER, G. & LOWENBERG, B. (1988). Stimulatory spectrum of human recombinant multi CSF (IL-3). *Blood*, **71**, 1609.

BRANDT, S.J., PETERS, W.P., ATWATER, S.K. & 7 others (1988). Effect of recombinant human granulocyte-macrophage colony-stimulating factor on haemopoietic reconstitution following high dose chemotherapy and autologous bone marrow transplantation. *N. Engl. J. Med.*, **318**, 869.

BRONCHUD, M.H., SCARFFE, J.H., THATCHER, N. & 5 others (1987). Phase I/II study of recombinant human granulocyte stimulating factor in patients receiving intensive chemotherapy for small cell lung cancer. *Br. J. Cancer*, **56**, 809.

CLARK, S.C. & KAMEN, R. (1987). The human haemotopoietic colony stimulating factors. *Science*, **236**, 1229.

COHEN, A.N., ZSEBO, K.B., INOUE, H. & 6 others (1987). *In vivo* stimulation of granulopoiesis by recombinant human granulocyte stimulating factor. *Proc. Natl Acad. Sci. USA*, **84**, 2484.

DEVEREUX, S., LINCH, D.C., CAMPOS-COSTA, D., SPITTLE, M.F. & JELLIFFE, A.M. (1987). Transient leucopenia induced by granulocyte-macrophage colony-stimulating factor (letter). *Lancet*, **ii**, 1523.

DEVEREUX, S., BULL, H.A., CAMPOS-COSTA, D., SAIB, R. & LINCH, D.C. (1988*a*). Granulocyte macrophage colony stimulating factor induced changes in cellular adhesion molecule expression and adhesion to endothelium: *In vitro* and *in vivo* studies in man. *Br. J. Haematol.* (in press).

DEVEREUX, S., LINCH, D.C., PATTERSON, K.P., GRIBBEN, J.G., McMILLAN, A. & GOLDSTONE, A.H.G., (1988*b*). GM–CSF accelerates neutrophil recovery after autologous bone marrow transplantation for Hodgkin's disease. *Bone Marrow Transplantation* (in press).

DE VITA, V.T. (1985). *Principles and Practice of Oncology*, 2nd edition, p. 266. Lippincott: Philadelphia.

DEXTER, T.M., ALLEN, T.D. & LAJTHA, L.G. (1977). Conditions controlling the proliferation of haemopoietic stem cells *in vitro*. *J. Cell. Physiol.*, **91**, 335.

DONAHUE, R.E., WANG, E.A., STONE, D.K. & 5 others (1987*a*). Stimulation of haematopoiesis in primates by continuous infusion of recombinant human GM–CSF. *Nature*, **321**, 872.

DONAHUE, R.E., SEEHRA, J., ROTON, C. & 7 others (1987*b*). Stimulation of haematopoiesis in primates with human interleukin-3 and granulocyte macrophage colony stimulating factor (abstr.). *Blood*, **70**, Suppl. 1, 388.

DURUM, S.K., SCHMIDT, J.A. & OPPENHEIM, J.J.S. (1985). Interleukin 1: An immunological perspective. *Ann. Rev. Immunol.*, **3**, 263.

GABRILOVE, J.L., JAKUBOWSKI, A., SCHER, H. & 11 others (1988). Effect of granulocyte colony stimulating factor on neutropaenia and associated morbidity due to chemotherapy for transitional cell carcinoma of the urothelium. *N. Engl. J. Med.*, **318**, 1414.

GROOPMAN, J.E., MITSUYASU, R.T., DeLEO, M.J., DETTE, D.H. & GOLDE, D.W. (1987). Effect of granulocyte-macrophage colony-stimulating factor on myelopoiesis in the acquired immune deficiency syndrome. *N. Engl. J. Med.*, **317**, 593.

KELLER, G., PAIGE, C., GILBOA, E. & WAGNER, E.F. (1985). Conditions controlling the proliferation of haemopoietic stem cells *in vitro*. *J. Cell. Physiol.*, **91**, 335.

MAYER, P., LAM, C., OBENAUS, H., LIEBL, E. & BESSENER, J. (1987). Recombinant human GM–CSF induces leukocytosis and activates peripheral blood polymorphonuclear neutrophils in non human primates. *Blood*, **70**, 206.

METCALF, D., BEGLEY, C.G., JOHNSON, G.R.M., NICOLA, N.A., LOPEZ, A.F. & WILLIAMSON, D.J. (1986). Effects of purified bacterially synthesised murine multi CSF (IL-3) on haematopoiesis in normal adult mice. *Blood*, **68**, 46.

METCALF, D., BEGLEY, C.G., WILLIAMSON, D.J. & 5 others (1987). Haemopoietic responses in mice injected with purified recombinant murine GM–CSF. *Exp. Haematol.*, **15**, 1.

MONROY, R.L., SKELLY, R.R., DAVIS, T.A. & 5 others (1987). Effect of interleukin-1 on the recovery of primates after radiation and autologous bone marrow transplantation. *Blood*, **70**, Suppl., 1103.

MOORE. M.A. & WARREN, D.J. (1987). Synergy of interleukin 1 and granulocyte colony stimulating factor: *In vivo* stimulation of stem cell recovery and haemopoietic regeneration following 5-fluorouracil treatment of mice. *Proc. Natl Acad. Sci. USA*, **84**, 7134.

MORSTYN, G., CAMPBELL, L., SOUZA, L.M. & 5 others (1988). Effect of granulocyte colony stimulating factor on neutropaenia induced by cytotoxic chemotherapy. *Lancet*, **i**, 667.

NETA, R., DOUCHES, S. & OPPENHEIM, J.J. (1986). Interleukin 1 is a radioprotector. *J. Immunol.*, **136**, 2483.

NIENHUIS, A.W., DONAHUE, R.E., KARRISON, S. & 7 others (1987). Recombinant human granulocyte-macrophage colony-stimulating factor shortens the period of neutropaenia after autologous bone marrow transplantation in a primate model. *J. Clin. Invest.*, **80**, 573.

PLUZNIK, D.H. & SACHS, L. (1965). The cloning of normal mast cells in cell culture. *J. Cell. Comp. Physiol.*, **66**, 319.

SIEFF, C.A., EMERSON, S.G., DONAHUE, R.E. & 5 others (1985). Human recombinant granulocyte-macrophage colony-stimulating factor: A human multilineage hemopoietin. *Science.*, **230**, 1171.

STANLEY, E.R., BARTOCCI, A., PATINKIN, D., ROSENDAAL, M. & BRADLEY, T.R. (1986). Regulation of very primitive, multipotent, hemopoietic cells by hemopoietin 1. *Cell*, **45**, 667.

STORK, L., KISSINGER, M. & ROBINSON, W. (1987). Interleukin-1 hastens murine granulocyte recovery following treatment with cyclophosphamide (abstr.). *Blood*, **70**, Suppl. 1, 808.

TILL, J.E. & MCCULLOCH, E.A. (1961). A direct measurement of the radiation sensitivity of normal mouse bone marrow cells. *Radiation Res.*, **14**, 213.

VADHAN-RAJ, S., KEATING, M., LEMAISTSRE, A & 5 others (1987). Effects of recombinant human granulocyte-macrophage colony-stimulating factor in patients with myelodysplastic syndromes. *N. Engl. J. Med.*, **317**, 1545.

WELTE, K., BONILLA, M.A., GILLIO, A.P. & 6 others (1987). Recombinant human granulocyte colony stimulating factor (G–CSF). *J. Exp. Med.*, **165**, 941.

WONG, G.C. & CLARK, S.C. (1988). Multiple actions of interleukin 6 within a cytokine network. *Immunol. Today*, **9**, 137.